# Characterization of Ablated Bone and Muscle for Long-Pulsed Laser Ablation in Dry and Wet Conditions

**DOI:** 10.3390/ma12081338

**Published:** 2019-04-24

**Authors:** Hervé Nguendon Kenhagho, Sergey Shevchik, Fatemeh Saeidi, Neige Faivre, Bastian Meylan, Georg Rauter, Raphael Guzman, Philippe Cattin, Kilian Wasmer, Azhar Zam

**Affiliations:** 1 Biomedical Laser and Optics Group, Department of Biomedical Engineering, University of Basel, Gewerbestrasse 14, 4123 Allschwil, Switzerland; 2 Laboratory for Advanced Materials Processing, Empa-Swiss Federal Laboratories for Materials Science and Technology, 3602 Thun, Switzerland; Sergey.Shevchik@empa.ch (S.S.); Fatemeh.Saeidi@empa.ch (F.S.); Neige.Faivre@empa.ch (N.F.); Bastian.Meylan@empa.ch (B.M.); Kilian.Wasmer@empa.ch (K.W.); 3 Bio-Inspired RObots for MEDicine-Lab, Department of Biomedical Engineering, University of Basel, Gewerbestrasse 14, 4123 Allschwil, Switzerland; georg.rauter@unibas.ch; 4 Department of Neurosurgery, University Hospital Basel, Spitalstrasse 21, 4056 Basel, Switzerland; Raphael.Guzman@usb.ch; 5 Center for medical Image Analysis and Navigation, Department of Biomedical Engineering, University of Basel, Gewerbestrasse 14, 4123 Allschwil, Switzerland; philippe.cattin@unibas.ch

**Keywords:** acoustic tissue response, laser ablation, tissue differentiation, influence of ablation condition

## Abstract

Smart laser technologies are desired that can accurately cut and characterize tissues, such as bone and muscle, with minimal thermal damage and fast healing. Using a long-pulsed laser with a 0.5–10 ms pulse width at a wavelength of 1.07 µm, we investigated the optimum laser parameters for producing craters with minimal thermal damage under both wet and dry conditions. In different tissues (bone and muscle), we analyzed craters of various morphologies, depths, and volumes. We used a two-way Analysis of Variance (ANOVA) test to investigate whether there are significant differences in the ablation efficiency in wet versus dry conditions at each level of the pulse energy. We found that bone and muscle tissue ablated under wet conditions produced fewer cracks and less thermal damage around the craters than under dry conditions. In contrast to muscle, the ablation efficiency of bone under wet conditions was not higher than under dry conditions. Tissue differentiation was carried out based on measured acoustic waves. A Principal Component Analysis of the measured acoustic waves and Mahalanobis distances were used to differentiate bone and muscle under wet conditions. Bone and muscle ablated in wet conditions demonstrated a classification error of less than 6.66% and 3.33%, when measured by a microphone and a fiber Bragg grating, respectively.

## 1. Introduction

Cutting tissue with drills and saws results in heat formation that may damage the surrounding tissues, leading to impaired bone regeneration [1,2,3]. In contrast, laser ablation (a laser used as an osteotomy tool) works without contact force, thereby reducing the severe mechanical vibrations and heat damage generated by conventional cutting tools [2,4]. Avoiding heat damage improves the healing of the cut bone, making laser ablation a highly desired innovation in the field of maxillo-facial-, neuro- and orthopedic surgery [2]. Moreover, lasers cut with greater precision than conventional tools [5,6,7]. When irradiating bone and teeth with the Er:YAG laser (2.94 μm), water and hydroxyapatite absorb most of the laser energy. Teeth (with a composition close to bone) consist of 85–95% carbonated hydroxyapatite, 8–12% water, and 2–3% protein and lipids [8,9,10]. Because the water content and Amide I/II bands in bone absorb so much IR radiation, different lasers for cutting bone in surgical applications have been investigated, including the holmium-doped yttrium aluminum garnet (Ho:YAG), carbon dioxide (CO_2_) lasers, the erbium-doped yttrium aluminum garnet (Er:YAG) pulsed laser, and free-electron lasers (FEL) [11,12,13,14,15,16,17]. The Er:YAG laser has a wavelength of 2.94 μm, corresponding to one of the absorption peaks of water and hydroxyapatite, the main component of bone [1,7], and corresponding to the wavelength where bone or teeth are ablated by means of photo-thermal vaporization.

The laser ablation process has been optimized for bone tissue and relies on a photothermic mechanism that results in thermal damage to the surrounding tissue unless a cooling system is used [18,19,20,21]. Recent investigations have shown that water on the exposed area prevents the carbonization of tissues around craters, thereby improving the ablation efficiency [22,23,24] and increasing the ablation rate [25,26]. It has been argued that a high laser energy density improves the radiation ablation efficiency in wet conditions by confining the laser-generated plasma in the liquid layer, leading to a greater emission of acoustic and shock waves as compared to ambient air conditions [26,27]. However, high-energy laser systems are not suitable for tissue ablation and regeneration under dry conditions, as they produce a greater temperature dissipation around the exposed zone, which can lead to carbonization and a long wound-recovery time.

Long-pulsed fiber lasers may be more practical in an operating room because of their fiber-coupled output, and their smaller footprint and robustness to environmental vibrations than short-pulsed lasers [28]. Long-pulsed lasers, such as millisecond-pulsed lasers, with several joules of energy, are also much less costly than ultra-short-pulsed lasers, such as femto or nanosecond pulsed ones, with millijoule energy. In addition, fiber lasers with a wavelength of 1.07 μm have a low absorption in water as compared to the Er:YAG lasers [29,30]. To minimize the energy loss in a wet environment, a laser wavelength of 1.07 µm may be appropriate as its absorption is lower than for the wavelength of the Er:YAG lasers [3]. Because the absorption coefficient for a fiber laser at 1.07 µm is low in water (approximately 0.4cm^−1^), the transmission through a 14 mm water layer is around 80% [27,31,32]. Therefore, when a bone is covered with water and irradiated by a fiber laser at 1.07 µm, it does not significantly reduce the energy reaching the bone. In contrast to a wavelength of 2.94 μm, this well-established wavelength for metal welding in the industry (1.07 µm) could be clinically studied for its suitability for knee surgery in a wet environment. Previous investigations were mainly conducted on metals and substrate samples for laser-welding applications [33,34].

However, in addition to being fast and accurate when cutting bone, lasers need to be safe as well. A tissue differentiation method could improve the safety of lasers as an osteotomy tool. This is particularly true if a laser system can be controlled by an *in situ* and real-time automatic feedback system that not only differentiates specific types of human tissues but additionally stops automatically when the laser encounters tissues that are not meant to be ablated [2,4,35]. Acoustic shock waves (ASWs) are generated when ablating material with a laser and can be measured using microphone and Fiber Bragg Grating (FBG) sensors [4,36,37,38]. Compared to standard microphones, FBG sensors are smaller, more sensitive, lightweight and immune to electromagnetic interference [39,40]. In this work, we investigate the laser tissue ablation of muscle and bone, using a long-pulsed laser with a center wavelength of 1.07 μm and pulse energies in the range of 0.75–15 J, under different ablation conditions (in terms of the ablation efficiency of bone and the impact of water on carbonization). We simultaneously recorded (with a microphone and an FBG) and differentiated the acoustic signals generated by the ablation of porcine bone and muscle. We used a Principle Component Analysis (PCA) to reduce the dimensionality of the ASWs measured from each specimen and the Mahalanobis distances method to differentiate the scores of the measured ASWs. Such a set-up has the potential to act as an optoacoustic feedback sensor for laser osteotomy.

## 2. Materials and Methods

### 2.1. Laser Tissue Ablation

**Figure 1:**
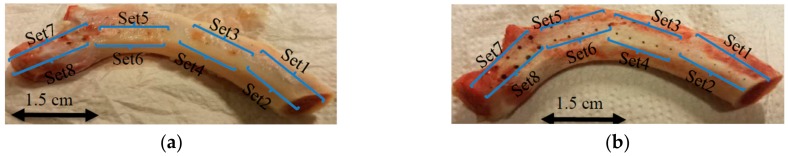
Ablated hard bone in (**a**) wet and (**b**) dry conditions at different pulse energies summarized in Table 1.

For the laser tissue ablation experiments, muscle and bone tissue specimens from a fresh porcine spare rib, purchased from a local slaughterhouse, were used. The bone and muscle were 10.80 and 11.00 mm thick, respectively. We ablated one specimen of the endosteum in the compact bone and muscle tissue. The laser ablation was performed at five different locations 4 mm apart on each specimen. Five craters exposed to ten laser pulses with the same laser parameters is referred to as a “Set”. Table 1 shows eight sets of experiments with pulse energies between 0.75 and 15 J. The experiments were conducted on different specimens in wet and dry conditions (see Figure 1 for the bone specimens). To perform the laser ablation under wet conditions, a spray of distilled water with a flow rate of 0.1 mL/s was directed to the ablation spot, to wet the specimen each time before a laser pulse hit the tissue. The dry condition was defined as the ablation without any distilled water sprayed at the ablation location before focusing the laser light. Laser-induced acoustic shock waves were measured during the ablation.

### 2.2. Experimental Set-Up

**Figure 2:**
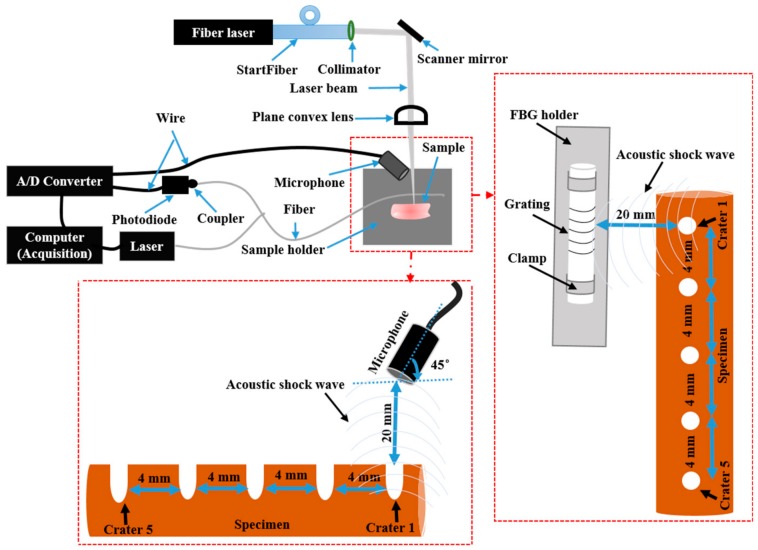
Schematic of the experiment illustrating the laser fiber set-up, an FBG and a microphone for laser-induced acoustic measurements.

A laser fiber source (StarFiber 150 P; ROFIN-LASAG AG, Belp, Switzerland) operates at 1.07 µm with a pulse repetition rate of 1 Hz and pulse duration at 0.5–10 ms. A scanner head (HurrySCAN 30; Scanlab GmbH, Puchheim, Germany) was used to precisely ablate the bone and muscle (Figure 2). The laser beam was transmitted through a single-mode optical fiber with a 12 μm core diameter and focused on the surface of the sample with a focusing lens (focal distance of 170 mm). This setup provided a spot size diameter of 30 µm (measured by a beam profilometer at a specific level of e^−2^) at the focal point, and a maximum pulse energy of 15 J. The acoustic shock wave that was generated during the ablation was measured simultaneously using a non-contact acoustic microphone: (PAC AM41 SNAA05, physical acoustics, Princeton Junction, NJ, USA) with −3 dB bandwidth of 39–42 kHz at a resonant frequency of 40 kHz and an FBG detection system (FFT Corning SMF-28, Corning, New York City, NY, USA) with a first grating position at 1 m, and an FBG length of 10 mm.

### 2.3. Analysis of Craters

#### 2.3.1. Characterization of Laser-Induced Craters

After the laser ablation process (in both the wet and dry conditions), the resulting holes/craters were characterized using a scanning electron microscope (SEM). The analysis of the bone and muscle surface was performed using a tungsten filament SEM (DSM 962; Carl Zeiss AG, Oberkochen, Germany) and a high-resolution field emission SEM (S-4800; Hitachi High-Technologies Corporation, Tokyo, Japan). The volume and depth of each crater was calculated based on 3D mapping, using a confocal microscope (VK-8700; Keyence Corporation, Osaka, Japan).

#### 2.3.2. Ablation Efficiency

To calculate the ablation efficiency, the depth and volume of the craters in the bone and muscle samples were averaged over the four best craters (the craters were collapsing, especially on the muscle surface, and the worst one was discarded.). The primary purpose of a two-way ANOVA is to understand if there is an interaction between the two independent variables on the dependent variable [41,42]. Hence, based on mean scores for the dependent variable across the two groups and factors (dry bone vs. wet bone and dry muscle vs. wet muscle), we used the two-way ANOVA test in the SPSS Statistics tool to check whether there are significant differences in the ablation efficiency between the wet and dry conditions at each pulse energy level (main effects for dependent variables). The irrigation condition (dry and wet) and energy level were considered as independent variables 1 and 2, respectively. The volume and depth were considered as dependent variables 1 and 2, respectively. When significant differences in the ablation efficiency were observed, we also investigated significant variables that showed a main effect using Bonferroni-corrected post hoc tests [43].

### 2.4. Characterization of Acoustic Shock Waves

**Figure 3:**
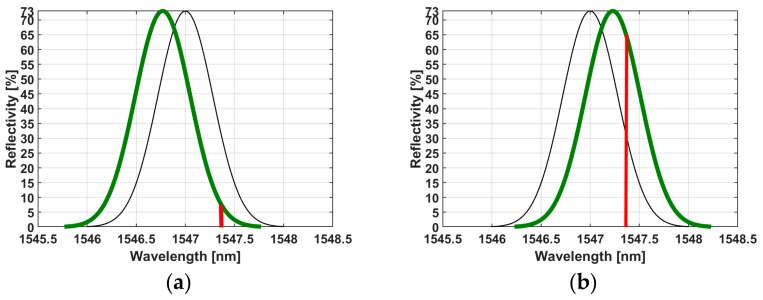
(**a**) Fiber in the compressive state (green) and resting state (black) with a tunable laser at a fixed wavelength of 1547.3 nm and 10% of the light reflected. (**b**) Fiber in the tension state (green) with a tunable laser at a fixed wavelength of 1547.3 nm and 65% of the light reflected.

To characterize the acoustic waves, we first determined the background noise level by measuring the environmental/floor noise using a microphone. The environmental noise was measured by switching off the ablation laser and measuring the acoustic signals in the room. Based on the measured floor noise, a signal/system detection was carried out using fixed amplitude thresholding with noise levels below 49 dB (0.28 mV). The fixed amplitude to trigger the measured acoustic signals was then set at 49 dB. To improve the sensitivity of the FBG, we clamped the fiber at both sides of the FBG holder. The FBG was positioned 2 cm away from the first ablated spot on the specimen (Figure 2). The calibrated microphone was fixed at a static position 2 cm away from the ablation spot at a 45° angle (approximately) to record the ASWs (Figure 2). Each sample (bone and muscle) and each condition (wet and dry), at the same location, was exposed to 10 laser pulses at a repetition rate of 1 Hz. The FBG was fixed with a constant strain, and the FBG read out was carried out at a wavelength of 1547.3 nm with a power of 2 mW. The spectral reflectance characteristics of our FBG sensor are shown in Figure 3a,b (black). According to the principles of the FBG operation [39,40], its structure is distorted by the coming Acoustic Emission (AE), thus shifting the reflectance window as depicted in Figure 3a,b (red). Under these conditions, the wavelength of the read out was chosen to fit the slope of the reflectance window, thus providing the linearly proportional response to the amplitude of the coming AE waves. The full dynamic range of the FBG shift, met in the experiments, provided a 10% and 65% reflectance, as is shown in Figure 3.

The detection of the reflected light was done using an InGaAs Switchable Gain Amplified Detector (PDA20CS-EC, Thorlabs, Ann Arbor, MI, USA), with a sensitivity in the spectral range of 800–1700 nm and 2.5 MHz of usable bandwidth. The photodiode detector senses the intensityvariation of the back-reflected light in the time domain, which is correlated to the acoustic signal generated during the laser ablation. The acoustic signal was transferred to the computer for recording and post-processing.

### 2.5. Tissue Differentiation

To differentiate hard bone and muscle, we looked at the ASW measured from the optimal ablation conditions. In this study, the optimum ablation was defined as the one that produced craters that were relatively clean from random charring and that showed fewer cracks compared to other craters in both conditions. The first twenty ASWs from the first two optimum craters were used as “training data”, while the last thirty ASWs from the last optimum three craters were considered “testing data”. To eliminate the phase shift effects at each measured ASW, we looked at the amplitude spectrum using the Fast Fourier Transform (FFT) in MATLAB (version R2016a). To reduce the dimensionality of each ASW, we used the amplitude spectrum as the input of the Principle Component Analysis (PCA). Then, we improved the contrast of the visualization of each ASW using the logarithm of the amplitude spectrum. To simultaneously differentiate the tissue types, we used the first three PCA scores from the set of training data combined with the Mahalanobis distance, and we plotted the 95% confidence ellipse using the three orthogonal eigenvectors of the scores from the “training data”. The scores from the testing data in each ellipsoid that correctly detected tissues were considered true positives; if they were outside the ellipsoid, the scores were considered unknown or false positive. We differentiated the tissues based on the ellipsoid because it considers the covariance of the ASW scores and the scales of the different variables. Therefore, it is useful for detecting members of the same group and even outliers.

## 3. Results

### 3.1. The Ablated Tissues at Different Morphological Craters

**Figure 4:**
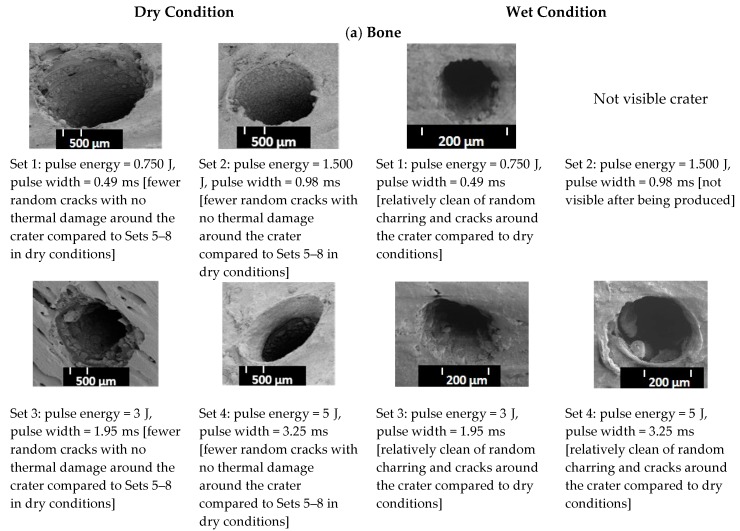

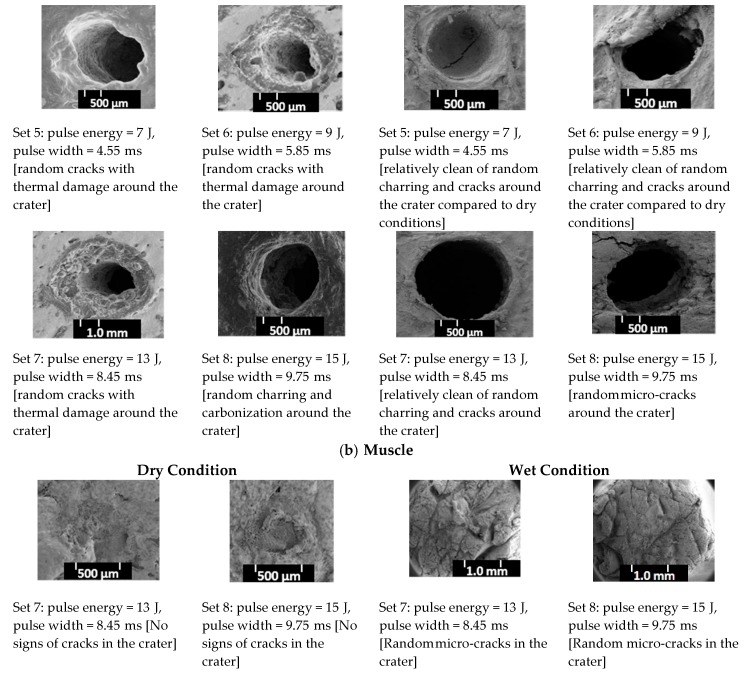
SEM top-views of (**a**) bone and (**b**) muscle surfaces in the dry and spray ablation after ten laser pulses.

**Figure 5:**
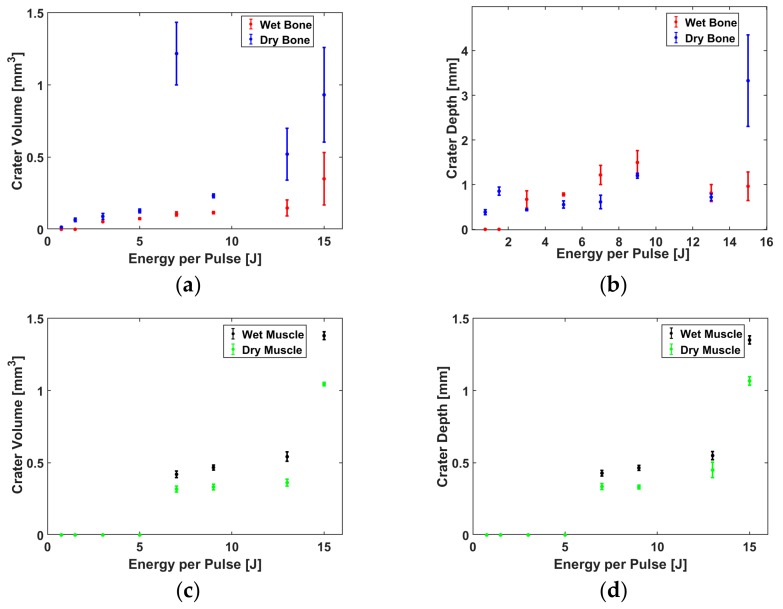
Bone and muscle comparison of the average ablation efficiency for five dry and wet craters as a function of energy with 10 pulses: (**a**) bone crater volume and (**b**) depth ablations; (**c**) muscle crater volume and (**d**) depth ablations.

Figure 4a,b shows the representative SEM pictures of the ablated bone and muscle surfaces, respectively, in dry and wet conditions for each set of conditions defined in Figure 4. In Figure 4a, the bones ablated in the dry conditions reveal an irregular surface with random propagating cracks at the surface for Sets 1 to 4. Sets 5 to 7 show irregular surfaces with thermal damage. The SEM picture of Set 8 shows a crater with random charring (around the crater as compared to the craters in Sets 1 to 7). For the ablation in wet conditions, Set 1 and Sets 3 to 7 indicate craters with less random charring, fewer cracks around the ablated contours and without any signs of thermal damage. Set 8 indicates some random micro cracks around the crater, setting an upper limit for the optimum laser bone ablation assisted by distilled water. In Figure 4b, the photographs of the muscle in dry and wet conditions in Sets 4 to 8 show no signs of a random star with thermal damage around the craters. It was not possible to reproduce the SEM pictures of the muscles in dry and wet conditions for Sets 1 to 6. During the drying procedure, at least 20% muscle shrinkage from the original state was observed. Sets 7 and 8 show signs of random cracks in the muscle crater under wet conditions only. Based on Figure 4 and Figure 5, we found that, at Set 8, we can ablate faster than for other sets, but we also burned tissues. The optimum ablation for hard bone and muscle was at Set 5 (wet conditions) because the craters were relatively clean of random charring and showed fewer cracks compared to other sets with higher energy in both conditions. Therefore, to see if we could also differentiate the ablated tissues using the measured ASWs, we decided to use the process parameters of Set 5 (7 J and 4.55 ms) in the wet condition.

### 3.2. Efficiency of Laser Bone and Muscle Ablation

The mean values and corresponding estimated marginal means with an error bar at a 95% confidence interval for volume and depth in bone and muscle are plotted in Figure 5. Based on the mean, the volume of the craters in bone under the dry conditions was higher than in the wet conditions, except when the pulse energy was set to 0.75 J (Figure 5a). The two-way ANOVA test (Appendix, Table A1) showed that differences in the mean volume of the craters created in the dry and wet conditions were statistically significant (*p* < 0.05, the *p*-value is labeled as “Sig.” in the SPSS output “Sig.” stands for the significance level). However, the difference in depth between the two conditions was not statistically significant (*p* > 0.05), (Appendix, Table A2). Using the Bonferroni-corrected post hoc tests, the volume showed a simple main effect between the dry and wet conditions at the energy levels of 13 and 15 J (*p* < 0.05), (Appendix, Table A3). In the muscle, Figure 5c,d shows no ablation until the pulse energy reaches 7 J. From 7 J, the depth and volume of the craters made in the muscle under the wet conditions were greater than those made in the dry conditions. Furthermore, the difference in the volume and depth of the muscle craters made in the dry versus wet conditions are statistically significant (*p* < 0.05), (Appendix, Table A4 and Table A5). From the Bonferroni-corrected post hoc tests, the volume and depth showed a simple main effect (a statistic difference) between the dry and wet conditions at each energy level (*p* < 0.05), (Appendix, Table A6 and Table A7).

### 3.3. Tissue Differentiation Based on Acoustic Measurement Using Microphone and FBG

**Figure 6:**
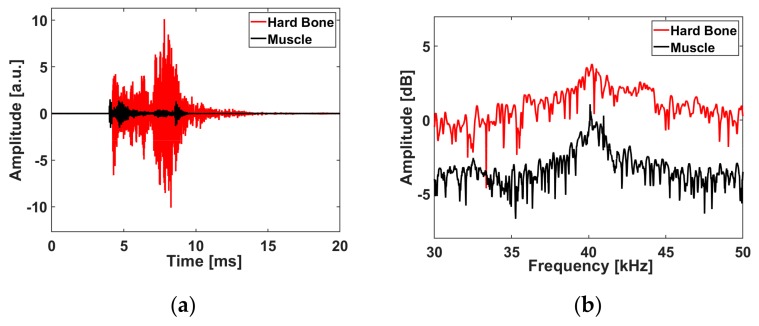

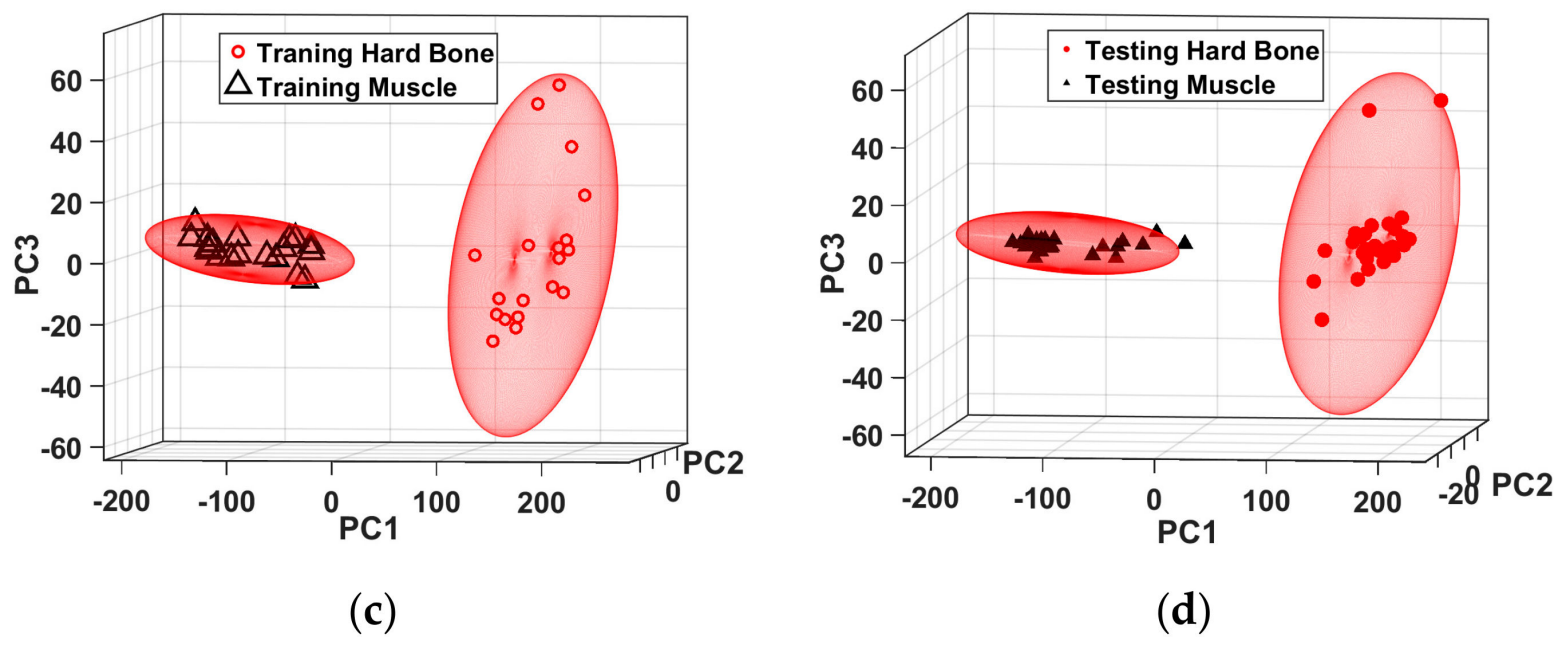
Acoustic shock wave differentiation measured by a microphone during laser pulse energy at 7 J in wet conditions (Set 5): (**a**) ASW in the time domain, (**b**) Spectrum of the ASW, (**c**) Ellipsoids based on 20 scores from training data, (**d**) Classification of 30 scores from the test data in each ellipsoid of the training data.

**Figure 7:**
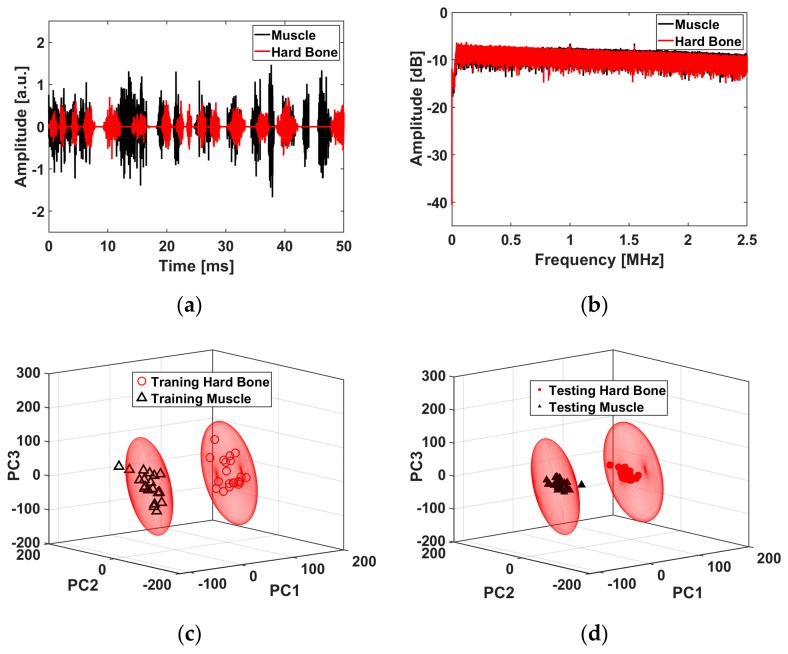
Acoustic shock wave differentiation measured by an FBG during laser ablation at 7 J in wet conditions (Set 5): (**a**) Back reflected light in the time domain, (**b**) Spectrum of the back-reflected light, (**c**) Ellipsoids based on 20 scores from training data, (**d**) Classification of 30 scores from the test data in each ellipsoid of the training data.

In Set 5 in the wet condition, the microphone measurements show that ten averaged acoustic signals for the wet-ablated muscle had a lower peak-to-peak amplitude in the time domain (Figure 6a) and a narrower amplitude spectrum than the wet-ablated bone (Figure 6b). In contrast, the peak-to-peak amplitude in the time domain (Figure 7a) and amplitude spectrum of the back-reflected light in the fiber was lower compared to those for the muscle (Figure 7b). The features that were chosen for the first three principle components (PC1, PC2, and PC3) of the PCA explained 99.91% and 22.70% of the variance in the acoustic waves recorded by the microphone and FBG, respectively. We plotted each ellipsoid against the three orthogonal eigenvectors of the training data scores to differentiate the bone and muscle (Figure 6c and Figure 7c). The scores from the testing data in each ellipsoid that correctly detected the tissues were considered as true positives; outside of the ellipsoid, the scores were considered as being unknown (Figure 6d and Figure 7d). From the confusion matrix, we see that the errors from the testing data (distinguishing between bone and muscle) were less than 6.66% for the ASWs measured by microphone (Table 2), and 3.33% for those measured by FBG (Table 3).

## 4. Discussion

Under the wet conditions, Sets 1 to 7 showed craters with less random charring, fewer cracks around the ablated contours and without any signs of thermal damage, as compared to the dry condition. This is because the water partially hydrates the exposed surface and thus partially prevents the carbonization of healthy tissue, leading to an improvement in the ablation efficiency of the muscle, as compared to the dry conditions (Figure 5c,d).

In contrast to the muscle, the volume of the craters made in the bone under the dry conditions was higher than those made in the wet conditions, except for a pulse energy of 0.75 J (Figure 5a). One possible reason for this outcome is that, in the absence of a water-cooling system, the pulse energy applied in the dry conditions causes more random cracks around the crater compared to those created in the wet conditions, thereby increasing the width and inducing a higher crater volume. In other words, the dry bone at the surface is ablated due to a higher temperature and possible phase transformation taking place in the wet conditions, induced by large temperature gradients inside the exposed material [4]. Apart from the crater volume in the bone created under the dry conditions, Figure 5b and the two-way ANOVA test (Appendix, Table A2) showed that the difference in the mean depth of the craters made in the dry versus wet conditions was not statistically significant (*p* > 0.05). This is due to the fact that bone rehydration was not sufficiently abundant to prevent an extensive heat diffusion [44]. In this experiment, the laser energy was increased by prolonging the laser pulse; with longer pulse durations, there was more time for the heat to diffuse and create carbonization at the bottom of the craters and saturate the depth of the craters. That is why Sets 4 to 8 presented some random cracks and thermal damage around the craters in the bone, created under the dry conditions. These phenomena are mainly caused by an excessive heat accumulation during and after an exposure to a high laser energy within the range of 7–15 J. Therefore, the range of the laser pulse duration, from approximately 0.5 to 10 ms, is likely to influence the amount of heat spread during the laser pulse [44,45]. The SEM pictures of the craters in the muscle for Sets 7 and 8, created in the dry conditions, presented no signs of random cracks around the craters.

However, when ablated under the wet conditions, these sets presented some signs of random cracks and the depths of the craters made in the muscle were lower than those in the bone (under both conditions). This is probably because the muscle tissue started to shrink directly after the ablation. In Set 5, we observed that the maximum amplitude of the acoustic signal efrom th bone ablated in the wet conditions was higher than that from the muscle. Muscle is composed of 79% water, while hard bone or teeth consist of 85–95% carbonated hydroxyapatite, 8–12% water, and 2–3% protein and lipids. Therefore, we believe that the carbonated hydroxyapatite component in bone produces greater amplitudes of sound, as it is a compact component compared to muscle, which is mostly made of water [8,9,10].

The resonant frequencies for bone and muscle cannot be used as parameters for tissue differentiation in the current stage of the project. This is mainly due to the limitation of the usable bandwidth (39–42 kHz) of the microphone that we used in this experiment. The spectrums of the acoustic pulses with shocks can extend beyond 1 MHz [46]. Thus, precise frequency measurements of broadband acoustic signals generated during ablation, using broadband pressure sensors, will measure acoustic shock waves with higher frequency components [35,47,48], which could then be used as frequency parameters to distinguish between ablated tissues. That is why the FBG, combined with a photodiode with a bandwidth of 2.5 MHz, resulted in a better classification error than the one measured by a microphone (Table 2 and Table 3).

In contrast to the microphone, the FBG measurements show that the bone tissues have a lower maximum amplitude of measured light than the muscle (Figure 7a,b). This was expected, as the higher amplitudes of ASWs from the bone might cause more distortion of the FBG, and more distortion creates more oscillation of the reflected light; thus, the measured reflected light intensity is low. The light intensity measured by the photodiode is reflected from the nonlinear regime of the near-Gaussian function (Figure 3a,b). Thus, the reflected light intensity measured by the photodiode is inferior during the ablation of the bone compared to the ablation of the muscle, which is situated in the linear regime. We measured the stretch and compression of the fiber that is converted into the measured intensity in the time domain (in real-time with an up to sub-nanosecond resolution) of the back-reflected light from the FBG structure. Consequently, the intensity is proportional to the compression strain on the FBG that is caused by acoustic shock waves.

## 5. Conclusions

The long-pulsed (from 0.5 to 10 ms) laser ablation of bone at a wavelength of 1.07 µm in wet and dry conditions was the focus of this study. The acoustic shock wave characteristics of bone and muscle during a laser ablation were also investigated in an attempt to differentiate between tissues. The tissue ablated with a spray irrigation produced very few cracks and thermal damage around the craters, which would ultimately lead to accelerated bone healing. For the bone and muscle tissue differentiation, we focused on the acoustic signal measured at Set 5 (7 J and 4.55 ms), under wet conditions. At this specific pulse energy, the mean depth of the crater in bone, created under wet conditions, was higher than that created in dry conditions; we also observed a lower ablation volume for craters created under wet conditions, compared to dry conditions. In Set 5, the craters in bone were relatively clean of random charring and showed fewer cracks than he craters made in higher energy sets in either condition. Keeping the ablation volume as small as possible, with less thermal damage and fewer cracks, could potentially improve the ablation efficiency and bone healing time. Using the best laser parameters (Set 5) to generate acoustic waves for tissue differentiation, the peak amplitude of the acoustic signals measured by a microphone for the bone was higher than the ones for muscle, in both dry and wet conditions. The classification error of the experiment, based on the spectral acoustic wave detection of bone and muscle in wet conditions, was less than 6.66% and 3.33%, as measured by a microphone and by FBG, respectively. By quantifying the measured acoustic shock waves, we guarantee an efficient tissue differentiation as feedback to reduce the probability of undesirable cutting of tissues at different depths and pulse energies.

The promising results of this approach motivate us for further improvements. Future work will include a histological study of the bones in a cross-section after the laser ablation, in order to fully evaluate the potentials of the technique in terms of the reduction of bone damage compared to other techniques. Furthermore, the advanced precision in signal differentiation, combined with an extension of the number of involved tissues and ablating regimes, needs to be investigated. This formulation leads to more complex data, and in order to reach efficiency in processing we plan to involve a cutting-edge machine learning technique, simultaneously providing a high temporal resolution and real-time operation of the methods involved. The challenges in this formulation reside in the contradiction between computational complexity, computational speed, and precision. The solution to this problem is planned as the continuation of this work.

## Figures and Tables

**Table 1:** Summary of the laser parameters.

Set	1	2	3	4	5	6	7	8
Pulse energy (J)	0.75	1.50	3	5	7	9	13	15
Pulse duration (ms)	0.49	0.98	1.95	3.25	4.55	5.85	8.45	9.75

**Table 2:** Confusion matrix for the bone and muscle classification during the laser ablation at 7 J pulse energy.

Tissue	Classified as			Classification Error %
	Bone	Muscle	Unknown	
Bone	30	0	1	3.33%
Muscle	0	28	2	6.66%

**Table 3:** Confusion matrix for the bone and muscle types during the laser ablation at 7 J pulse energy.

Tissue	Classified as			Classification Error %
	Bone	Muscle	Unknown	
Bone	30	0	0	0%
Muscle	0	30	0	3.33%

## Appendix

**Table A1:** Tests of Between-Subjects Effects.

Dependent Variable: Volume						
Source	Type III Sum of Squares	df	Mean Square	F	Sig.	Partial Eta Squared
Corrected Model	7.457 ^a^	15	0.497	8.528	0.000	0.727
Intercept	4.045	1	4.045	69.384	0.000	0.591
Irrigation Condition	1.370	1	1.370	23.506	0.000	0.329
Energy Level	3.990	7	0.570	9.778	0.000	0.588
Irrigation Condition * Energy Level	2.097	7	0.300	5.138	0.000	0.428
Error	2.798	48	0.058			
Total	14.300	64				
Corrected Total	10.255	63				

Based on estimated marginal means. * The mean difference is significant at the 0.05 level. ^a^ R Squared = 0.727 (Adjusted R Squared = 0.642).

**Table A2:** Tests of Between-Subjects Effects.

Dependent Variable: Depth						
Source	Type III Sum of Squares	df	Mean Square	F	Sig.	Partial Eta Squared
Corrected Model	35.387 ^a^	15	2.359	6.544	0.000	0.672
Intercept	49.438	1	49.438	137.127	0.000	0.741
Irrigation Condition	1.129	1	1.129	3.133	0.083	0.061
Energy Level	21.608	7	3.087	8.562	0.000	0.555
Irrigation Condition * Energy Level	12.650	7	1.807	5.012	0.000	0.422
Error	17.305	48	0.361			
Total	102.131	64				
Corrected Total	52.693	63				

Based on estimated marginal means. * The mean difference is significant at the 0.05 level. ^a^ R Squared = 0.672 (Adjusted R Squared = 0.569).

**Table A3:** Bonferroni-corrected post hoc tests (Irrigation Condition * Energy Level).

Dependent Variable: Volume							
Energy Level	(I) Irrigation Condition	(J) Irrigation Condition	Mean Difference (I-J)	Std. Error	Sig. ^b^	95% Confidence Interval for Difference ^b^	
						Lower Bound	Upper Bound
0.75 J	Dry	Wet	0.015	0.171	0.932	−0.329	0.358
	Wet	Dry	−0.015	0.171	0.932	−0.358	0.329
1.5 J	Dry	Wet	0.065	0.171	0.703	−0.278	0.409
	Wet	Dry	−0.065	0.171	0.703	−0.409	0.278
3 J	Dry	Wet	0.037	0.171	0.829	−0.306	0.380
	Wet	Dry	−0.037	0.171	0.829	−0.380	0.306
5 J	Dry	Wet	0.041	0.171	0.809	−0.302	0.385
	Wet	Dry	−0.041	0.171	0.809	−0.385	0.302
7 J	Dry	Wet	1.111 *	0.171	0.000	0.768	1.455
	Wet	Dry	−1.111 *	0.171	0.000	−1.455	−0.768
9 J	Dry	Wet	0.116	0.171	0.499	−0.227	0.460
	Wet	Dry	−0.116	0.171	0.499	−0.460	0.227
13 J	Dry	Wet	0.373 *	0.171	0.034	0.030	0.717
	Wet	Dry	−0.373 *	0.171	0.034	−0.717	−0.030
15 J	Dry	Wet	0.582 *	0.171	0.001	0.238	0.925
	Wet	Dry	−0.582 *	0.171	0.001	−0.925	−0.238

Based on estimated marginal means. * The mean difference is significant at the 0.05 level. ^b^ Adjustment for multiple comparisons: Least Significant Difference (equivalent to no adjustments).

**Table A4:** Tests of Between-Subjects Effects.

Dependent Variable: Volume						
Source	Type III Sum of Squares	df	Mean Square	F	Sig.	Partial Eta Squared
Corrected Model	4.268 ^a^	7	0.610	282.055	0.000	0.988
Intercept	11.846	1	11.846	5480.688	0.000	0.996
Irrigation Condition	0.283	1	0.283	130.989	0.000	0.845
Energy Level	3.921	3	1.307	604.659	0.000	0.987
Irrigation Condition * Energy Level	0.064	3	0.021	9.806	0.000	0.551
Error	0.052	24	0.002	-	-	-
Total	16.166	32	-	-	-	-
Corrected Total	4.319	31	-	-	-	-

Based on estimated marginal means. * The mean difference is significant at the 0.05 level. ^a^ R Squared = 0.988 (Adjusted R Squared = 0.984).

**Table A5:** Tests of Between-Subjects Effects.

Dependent Variable: Depth						
Source	Type III Sum of Squares	df	Mean Square	F	Sig.	Partial Eta Squared
Corrected Model	4.119 ^a^	7	0.588	259.632	0.000	0.987
Intercept	11.834	1	11.834	5220.932	0.000	0.995
Irrigation Condition	0.263	1	0.263	115.947	0.000	0.829
Energy Level	3.810	3	1.270	560.325	0.000	0.986
Irrigation Condition * Energy Level	0.046	3	0.015	6.833	0.002	0.461
Error	0.054	24	0.002	-	-	-
Total	16.008	32	-	-	-	-
Corrected Total	4.174	31	-	-	-	-

Based on estimated marginal means. * The mean difference is significant at the 0.05 level. ^a^ R Squared = 0.987 (Adjusted R Squared = 0.983).

**Table A6:** Bonferroni-corrected post hoc tests (Irrigation Condition * Energy Level).

Dependent Variable: Volume							
Energy Level	(I) Irrigation Condition	(J) Irrigation Condition	Mean Difference (I-J)	Std. Error	Sig. ^b^	95% Confidence Interval for Difference ^b^	
						Lower Bound	Upper Bound
7 J	Dry	Wet	−0.103 *	0.033	0.005	−0.170	−0.035
	Wet	Dry	0.103 *	0.033	0.005	0.035	0.170
9 J	Dry	Wet	−0.135 *	0.033	0.000	−0.203	−0.067
	Wet	Dry	0.135 *	0.033	0.000	0.067	0.203
13 J	Dry	Wet	−0.180 *	0.033	0.000	−0.248	−0.112
	Wet	Dry	0.180 *	0.033	0.000	0.112	0.248
15 J	Dry	Wet	−0.335 *	0.033	0.000	−0.403	−0.267
	Wet	Dry	0.335 *	0.033	0.000	0.267	0.403

Based on estimated marginal means. * The mean difference is significant at the 0.05 level. ^b^ Adjustment for multiple comparisons: Least Significant Difference (equivalent to no adjustments).

**Table A7:** Bonferroni-corrected post hoc tests (Irrigation Condition * Energy Level).

Dependent Variable: Depth							
Energy Level	(I) Irrigation Condition	(J) Irrigation Condition	Mean Difference (I-J)	Std. Error	Sig. ^b^	95% Confidence Interval for Difference ^b^	
						Lower Bound	Upper Bound
7 J	Dry	Wet	−0.127 *	0.034	0.001	−0.197	−0.058
	Wet	Dry	0.127 *	0.034	0.001	0.058	0.197
9 J	Dry	Wet	−0.135 *	0.034	0.001	−0.204	−0.066
	Wet	Dry	0.135 *	0.034	0.001	0.066	0.204
13 J	Dry	Wet	−0.150 *	0.034	0.000	−0.219	−0.081
	Wet	Dry	0.150 *	0.034	0.000	0.081	0.219
15 J	Dry	Wet	−0.313 *	0.034	0.000	−0.382	−0.243
	Wet	Dry	0.313 *	0.034	0.000	0.243	0.382

Based on estimated marginal means. * The mean difference is significant at the 0.05 level. ^b^ Adjustment for multiple comparisons: Least Significant Difference (equivalent to no adjustments).
